# Feasibility of one-stop multi-energy dynamic photon-counting perfusion CT for resectable HCC evaluation: lesion detection and histopathological correlations

**DOI:** 10.1186/s13244-026-02462-7

**Published:** 2026-11-04

**Authors:** Huimin Lin, Jiahao Zhou, Rong Deng, Zhihan Xu, Yuchen Yang, Yongjun Chen, Huanhuan Chong, Miao Ruan, Chunxue Yang, Haipeng Dong, Rui Chang, Ruokun Li, Weixia Li, Fuhua Yan

**Affiliations:** 1 Department of Radiology, Ruijin Hospital, Shanghai Jiao Tong University School of Medicine, Shanghai, China; 2 Faculty of Medical Imaging Technology, College of Health Science and Technology, Shanghai Jiao Tong University School of Medicine, Shanghai, China; 3 CT Collaboration, Siemens Healthineers, Shanghai, China; 4 Department of General Surgery, Ruijin Hospital, Shanghai Jiao Tong University School of Medicine, Shanghai, China; 5 Department of Pathology, Ruijin Hospital, Shanghai Jiao Tong University School of Medicine, Shanghai, China

**Keywords:** Multidetector computed tomography, Perfusion imaging, Carcinoma (hepatocellular), Neoplasm grading, Neoplasm invasiveness

## Abstract

**Objectives:**

To assess the feasibility of one-stop multi-energy dynamic volume perfusion photon-counting CT (VP-PCCT) for hepatocellular carcinoma (HCC) evaluation, including lesion detection and correlations with histopathology.

**Materials and methods:**

From July 2024 to June 2025, 407 participants with suspected malignant liver lesions prospectively underwent VP-PCCT, and 177 patients with pathology-confirmed HCC who received three-phase energy-integrating detector CT (EID-CT) were retrospectively enrolled for comparisons of image quality and lesion detectability. Single-phase images reconstructed from VP-PCCT perfusion datasets were compared with EID-CT. Perfusion parameters were analyzed between groups stratified separately by histological grade and microvascular invasion (MVI), and logistic regression was employed to identify independent indicators.

**Results:**

Ninety-two participants [median age, 62 years (IQR, 53–69); 76 males] with pathology-confirmed HCC were included in the VP-PCCT group and matched with 92 in the EID-CT group. Compared with EID-CT, 40-keV VP-PCCT provided comparable image quality for HCC and vessels with a similar tumor detection rate but a lower radiation dose. Poorly-differentiated HCCs showed higher tumor hepatic perfusion index (HPI_tumor_) and peritumoral blood flow (BF_peritumor_); MVI-positive HCCs exhibited lower tumor arterial liver perfusion (ALP_tumor_) and normalized blood flow of peritumoral region (BF_norm_peritumor_) (all *p* < 0.05). HPI_tumor_ [odds ratio (OR): 1.06] and BF_peritumor_ (OR: 1.04) were independent risk factors for poorly differentiated HCC, whereas decreased ALP_tumor_ (OR: 0.97) and BF_norm_peritumor_ (OR: 0.25) were associated with MVI positivity (all *p* < 0.05).

**Conclusions:**

One-stop VP-PCCT delivers high-quality morphologic and perfusion evaluation in a single low-dose scan, with hemodynamic profiles that may be associated with histological grade and MVI status.

**Key Points:**

***Question*** Clinical use of dynamic volume perfusion CT for comprehensively evaluating HCC without additional multiphase contrast-enhanced imaging remains limited.

***Findings*** Multi-energy dynamic photon-counting perfusion CT provided high-quality morphologic imaging of HCC at a low dose, with perfusion parameters independently indicating poorly differentiated HCC and MVI.

***Critical relevance statement*** One-stop multi-energy dynamic photon-counting perfusion CT enables high-quality morphologic and functional assessment in a single low-dose scan, providing comprehensive HCC evaluation with hemodynamic profiling that may support the assessment of histological grade and MVI.

**Graphical Abstract:**

**Figure:**
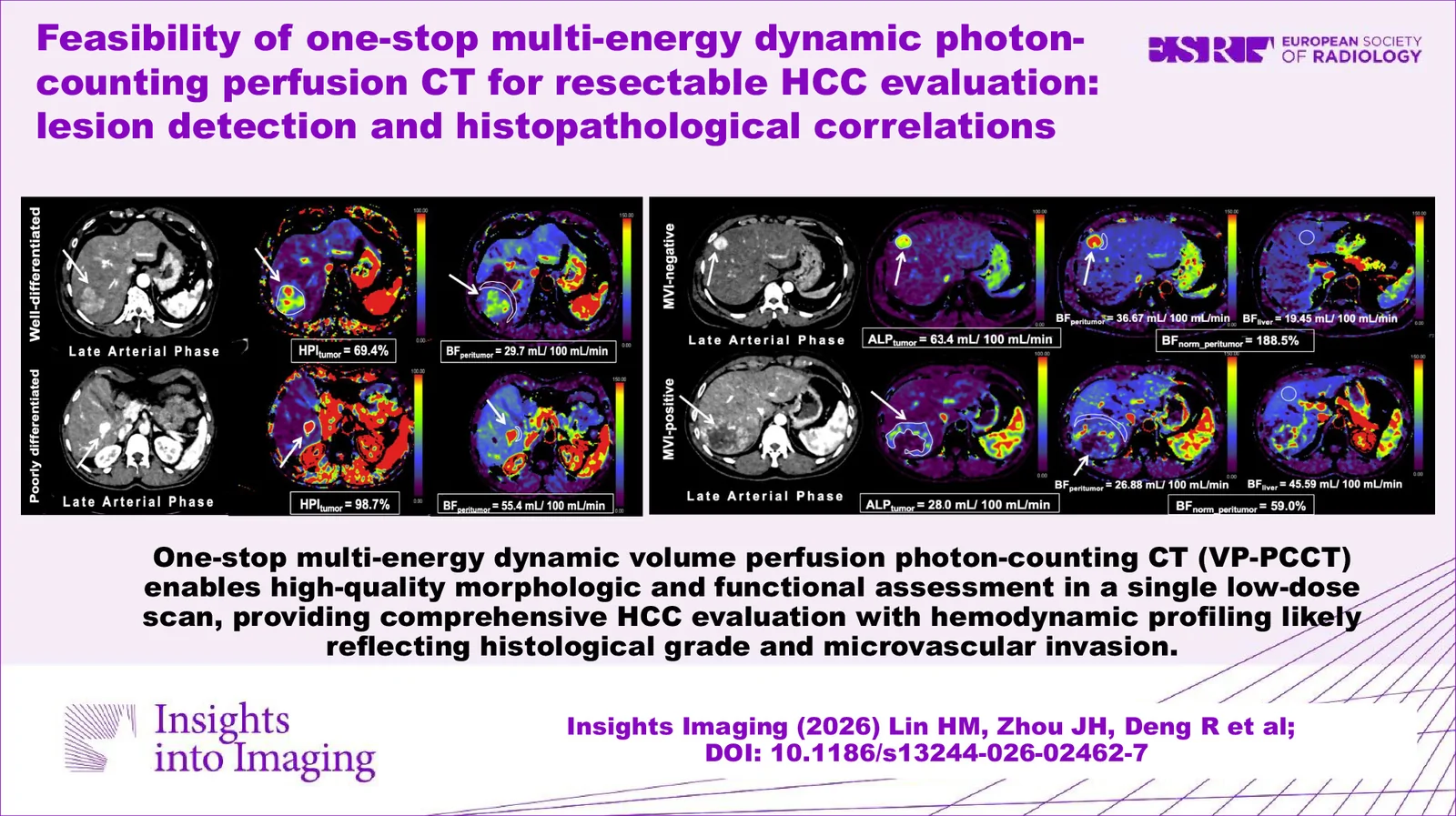

## Introduction

As the most common primary hepatic malignancy, hepatocellular carcinoma (HCC) is a leading cause of cancer death worldwide, exhibiting heterogeneous recurrence risk and treatment response [1]. Diagnosis depends on noninvasive imaging; however, histologic features, such as tumor grade and microvascular invasion (MVI), which strongly predict tumor aggressiveness and prognosis, are unavailable during treatment planning. To address this, various imaging features, including large tumor size, arterial peritumoral enhancement, and nonsmooth margins, have been proposed as indicators [2], although their qualitative nature and high interobserver variability limit their clinical value.

During HCC neoangiogenesis, decreased portal inflow and increased arterial supply lead to characteristic arterial-phase hyperenhancement with subsequent portal- or delayed-phase wash-out [3, 4]. Dynamic volume perfusion CT (VPCT) provides quantitative insights into the microcirculation of liver parenchyma and focal lesions, making it a promising method for HCC diagnosis and progression assessment [5]. However, conventional energy-integrating detector (EID)-based VPCT has not been widely adopted owing to its high radiation dose, limited scan coverage, and lack of standardized protocols, with dose burden considered the major barrier. To minimize radiation exposure, techniques with lower tube voltage and/or reduced tube current may be applied, but such strategies often increase image noise and beam-hardening artifacts, impairing perfusion quantification and image evaluation [6, 7]. In such cases, additional multiphase CT or mean temporal images can enhance morphological assessment without improving quantitative accuracy.

Photon-counting detector CT (PCCT) is increasingly being employed in clinical practice. Unlike energy-integrating detector CT (EID-CT), PCCT directly counts individual X-ray photons and sorts them into energy bins. This feature enhances dose efficiency, improves contrast resolution, and eliminates electronic noise [8, 9]. In abdominal imaging, PCCT reduces radiation dose by > 50% compared with EID-CT [10], and threshold-based noise reduction further improves image quality in photon-starved regions [11]. In particular, PCCT’s superior contrast resolution benefits liver tumor evaluation, enabling precise lesion delineation [12, 13]. Additionally, flexible low-keV virtual monoenergetic image (VMI) reconstruction further enhances tissue contrast, facilitating accurate lesion evaluation [14]. These characteristics make PCCT well-suited for VPCT, allowing combined high-resolution morphological and functional assessment in a single low-dose examination.

This study aimed to determine the feasibility of one-stop multi-energy dynamic VPCT with PCCT (VP-PCCT) for HCC by comparing image quality and radiation dose with multiphase EID-CT and assessing perfusion parameters as biomarkers for tumor histological grade and MVI status.

## Materials and methods

This prospective observational study was approved by the institutional review board of Ruijin Hospital (approval no. KY2024–187), with written informed consent obtained from all participants. One author is an employee of Siemens but did not have access to the study data. All other authors had full control of the data.

### Study population

**Fig. 1:**
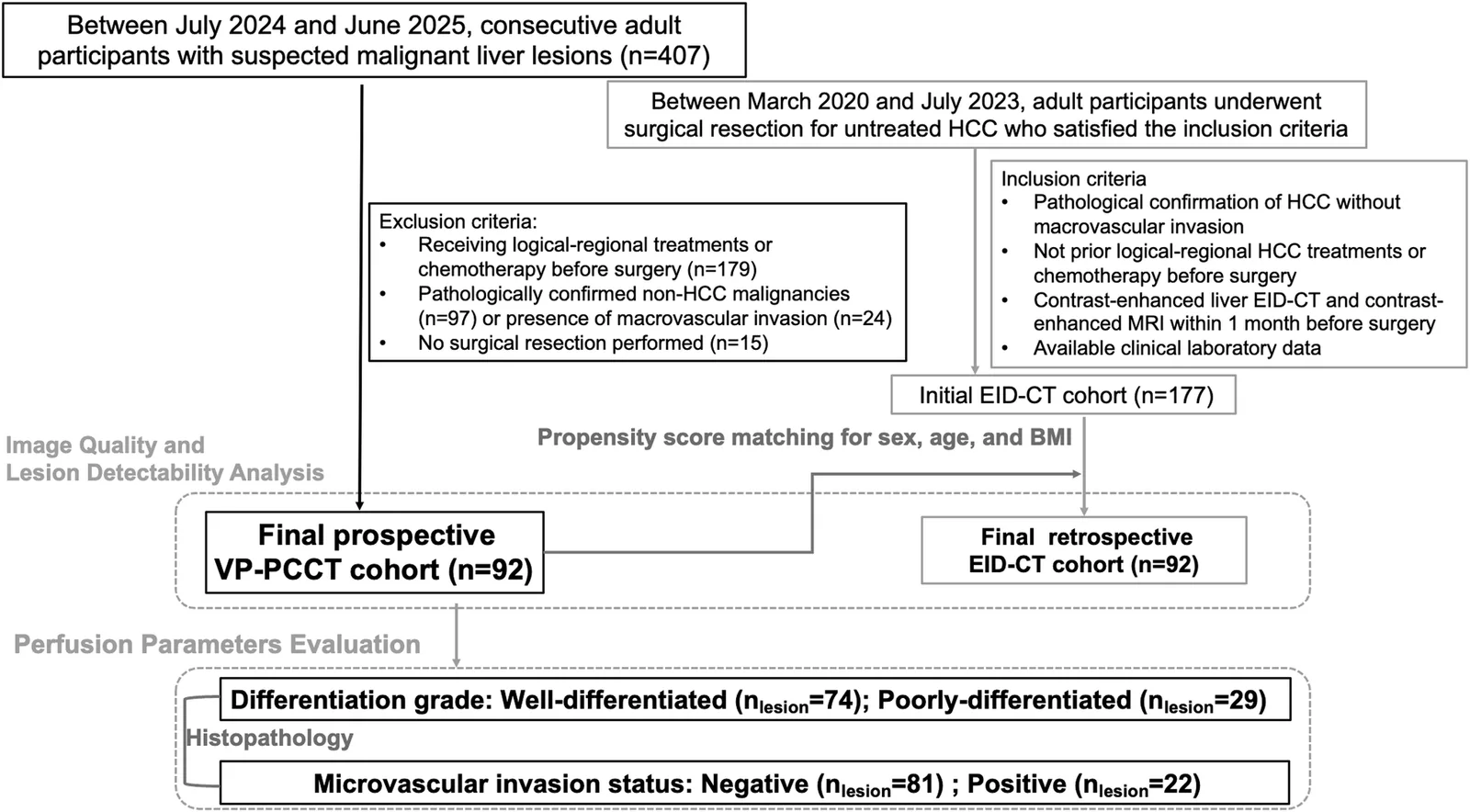
Flowchart of study population enrollment. HCC, hepatocellular carcinoma; VP-PCCT, dynamic volume perfusion CT with photon-counting CT; EID-CT, energy-integrating detector CT

During July 2024–June 2025, 407 adult participants with suspected malignant liver lesions were prospectively recruited for VP-PCCT and MRI (reference standard for lesion count). During March 2020–July 2023, 177 adult participants with pathologically confirmed HCC who underwent EID-CT were retrospectively recruited for comparisons of image quality and lesion detectability. Propensity score matching (PSM) was employed to balance potential confounders. Inclusion and exclusion criteria are provided in Fig. 1 and Appendix S1. Recorded demographic and clinical data included age, sex, body mass index (BMI), chronic hepatitis history, Barcelona clinic liver cancer stage, Child–Pugh grade, and preoperative laboratory tests.

### Scan protocols

VP-PCCT was performed using a first-generation, dual-source PCCT system (NAEOTOM Alpha, software version VA50; Siemens Healthineers) in adaptive, four-dimensional spiral mode. Scanning parameters were as follows: 90 kVp, 16-cm scan range, and automated tube current modulation (CareDose 4D; image quality level 50). In total, 40 mL of contrast medium (Iomeron, 400 mgI/mL; Bracco) was injected at 4–5 mL/s, followed by 30 mL of saline at the same rate. Scanning began 8 s post-injection, with 26 scans (20 and 6 at 1.4 and 2.8 s intervals, respectively) acquired over 43.4 s. Images were reconstructed with quantum iterative reconstruction level 3 and Br40 kernel, using 1-mm slices for individual phases (four VMIs at 40, 50, 55, and 60 keV) and 5-mm slices for perfusion analysis (T3D).

Retrospective EID-CT was performed using three scanners (Philips Healthcare). Each participant underwent standardized contrast-enhanced liver MRI. Parameters are detailed in Appendices S2–S3 and Table S1.

### PCCT image postprocessing

A dedicated workstation (Syngo.Via, version VB60; Siemens Healthineers) was used for image post-processing. Individual noncontrast, late arterial, and portal venous phases were extracted from VP-PCCT via the Dynamic Angio application. Perfusion maps were generated using commercial software (Body Perfusion, Siemens Healthineers). Perfusion parameters, including blood flow (BF; mL/100 mL/min), blood volume (BV; mL/100 mL), flow extraction product (FEP; mL/100 mL/min), arterial liver perfusion (ALP; mL/100 mL/min), portal venous perfusion (PVP; mL/100 mL/min), and hepatic perfusion index (HPI; %) were calculated using deconvolution and dual-input maximum-slope modes. Further details are provided in Appendix S4.

### Image quality assessment, lesion detectability, and radiological feature evaluation

CT images were independently reviewed by two blinded readers (H.L. and J.Z., with 10 and 6 years of abdominal imaging experience, respectively), who were unaware of clinical history, radiologic reports, and pathology. Using MRI, HCC lesion counts were defined by a third expert (F.Y., 37 years of experience), who also resolved any feature assessment discrepancies.

Image quality and lesion detectability were assessed for VP-PCCT and EID-CT. In VP-PCCT, VMI optimization was randomly tested in 20 participants, with the optimal level applied to the entire cohort for EID-CT comparisons. Image noise, signal-to-noise ratio (SNR), and contrast-to-noise ratio (CNR) were objectively analyzed for HCC, liver parenchyma, and vessels. Subjective evaluation (five-point scale) assessed image noise, vessel sharpness, and lesion conspicuity of HCC (Appendix S5). Lesion detectability was based on three-phase images for EID-CT and on three-phase images plus HPI maps for VP-PCCT. The HCC detection rate was defined as the number of CT-detected lesions relative to MRI-detected lesions.

**Fig. 2:**
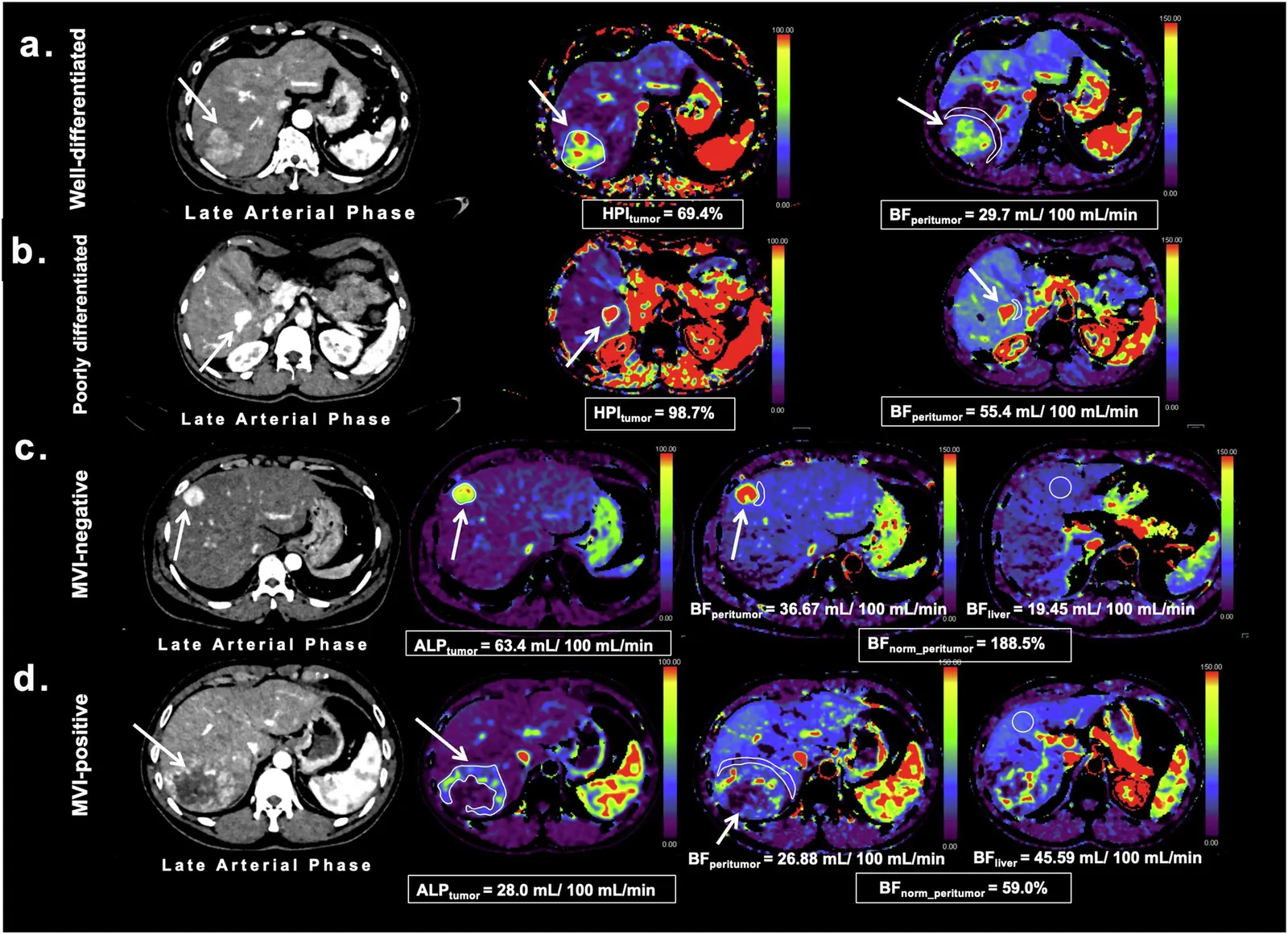
VP-PCCT perfusion maps and corresponding late arterial phase images demonstrate perfusion differences in HCC stratified by histological grade and MVI status. **a**, **b** Representative color-coded maps and corresponding late arterial phase images of HPI and BF in a 67-year-old man with well-differentiated HCC (**a**) and a 55-year-old man with poorly-differentiated HCC (**b**). **c**, **d** Maps and late arterial phase images of ALP and BF in a 41-year-old man with MVI-negative HCC (**c**) and a 38-year-old man with MVI-positive HCC (**d**). White arrows indicate tumor locations; contours delineate ROIs for tumor, peritumoral region, and liver parenchyma. Subscripts indicate measurement regions: _tumor = tumor, _peritumor = peritumoral region, _liver = liver parenchyma, _norm_peritumor = percentage of peritumoral to corresponding liver parenchymal perfusion values. HCC, hepatocellular carcinoma; MVI, microvascular invasion; VP-PCCT, dynamic volume perfusion CT with photon-counting CT

Five radiologic features were evaluated using VP-PCCT images: tumor count, maximum tumor diameter, peritumoral enhancement, tumor margin, and pseudocapsule. Perfusion parameters were assessed for tumor, peritumoral region, and liver parenchyma. Example images and measurement details are provided in Fig. 2 and Appendix S6.

### Histopathologic examination

Two pathologists (C.Y. and M.R., with 4 and 10 years of pathology experience, respectively) reviewed all surgical specimens. Histopathologic features included Edmondson–Steiner differentiation grade, MVI presence, and surrounding nontumoral liver inflammation grade and fibrosis stage (Scheuer system). Edmondson–Steiner grades I–II were classified as well-differentiated tumors, whereas grades III–IV were classified as poorly differentiated tumors [15].

### Statistical analysis

Normality was assessed using the Shapiro–Wilk test. Continuous variables are presented as means ± standard deviations or medians with interquartile ranges (IQRs), whereas categorical variables are shown as counts and percentages.

PSM (1:1 nearest neighbor) balanced age, gender, and BMI between the VP-PCCT and EID-CT groups. Optimal VMI selection in VP-PCCT was determined using the Friedman test to compare objective and subjective scores across VMIs, followed by Benjamini–Hochberg-corrected post-hoc testing. Group differences in clinical characteristics and image quality were assessed using Student’s *t*-test or the Mann–Whitney *U*-test, and the χ² test or Fisher’s exact test, as appropriate. The χ² test was also applied to compare HCC detection rates between groups.

Interobserver agreement for perfusion measurements was assessed using the intraclass correlation coefficient (ICC). Perfusion parameters stratified by pathology were compared via the Mann–Whitney *U*-test. Logistic regression was performed to identify independent perfusion indicators of HCC grade and MVI status, with variables showing *p* < 0.1 in univariable analysis entered into multivariable models. Backward selection was employed, and results were reported as odds ratios (ORs) with corresponding 95% confidence intervals (CIs). Analyses were performed using R (v4.1.0; 2021), with two-sided *p* < 0.05 considered significant.

## Results

### Participant characteristics

Of 407 participants who underwent VP-PCCT, 92 were eligible for analysis [median age: 62.0 years (IQR: 53.0–69.0); 76 males (82.6%)]. The remaining 315 were excluded, following the study criteria (Fig. 1, Appendix S7). In the retrospective EID-CT group, 92 patients were included following PSM for sex, age, and BMI. The VP-PCCT group’s demographics are shown in Table 1; those of the matched EID-CT group are provided in Table S2. The median dose–length product [884 mGy·cm (IQR, 756–1028) vs 934 mGy·cm (IQR, 791–1127), *p* = 0.03] and the effective dose [13.3 mSv (IQR, 11.3–15.4) vs 14.0 mSv (IQR, 11.9–16.9); *p* = 0.03] were lower with VP-PCCT than with EID-CT.

**Table 1:** Clinical and pathologic characteristics of the VP-PCCT group

Characteristic	VP-PCCT group (*n* = 92)
Age (y)^*^	62 (53–69)
BMI (kg/m^2^)^*^	23.4 (22.0–26.0)
Sex	
Male	76 (82.6)
Female	16 (17.4)
Etiology of liver disease	
Chronic hepatitis B/C	70 (76.1)
Others	22 (23.9)
Steatosis	4 (4.3)
Primary biliary cholangitis	1 (1.1)
Alcoholic cirrhosis	1 (1.1)
Normal	16 (17.4)
Child–Pugh classification	
A	90 (97.8)
B	2 (2.2)
BCLC stage	
Stage 0/A	84 (91.3)
Stage B	8 (8.7)
Laboratory findings	
AFP level	
≤ 200 ng/mL	78 (84.8)
> 200 ng/mL	14 (15.2)
ALT level	
≤ 50 U/L	69 (75.0)
> 50 U/L	23 (25.0)
AST level	
≤ 40 U/L	59 (64.1)
> 40 U/L	33 (35.9)
Tumor number per participant	
solitary	83 (90.2)
multiple	9 (9.8)
Tumor size	
≤ 5 cm	69 (75.0)
> 5 cm	23 (25.0)
Time interval^*^	
CT–MRI (day)	1 (1–6)
CT–Surgery (day)	2 (2–4)
Pathologic findings	
Edmonson–Steiner grade (n_lesion_=103)^‡^	
I–II	74 (71.8)
III–IV	29 (28.2)
MVI (*n*_lesion_ = 103)^‡^	
Negative	81 (78.6)
Positive	22 (21.4)
Inflammation grade of liver parenchyma	
G1–2	63 (68.5)
G3–4	29 (31.5)
Fibrosis stage of liver parenchyma	
S1–2	37 (40.2)
S3–4	55 (59.8)

Unless otherwise indicated, data are numbers of patients or tumors, and data in parentheses are percentages

*VP-PCCT* dynamic volume perfusion CT with photon-counting CT, *BCLC* Barcelona Clinic Liver Cancer, *AFP* α-fetoprotein, *ALT* alanine aminotransferase, *AST* aspartate aminotransferase

^*^ Data are medians, with interquartile ranges in parentheses

^‡^ Number of lesions

Statistically significant *p* < 0.05 values are in bold

### Participant-based comparison of image quality and detection rate between the VP-PCCT and EID-CT groups

VMI optimization was randomly tested in 20 participants in VP-PCCT. Among monoenergetic images at 40–60 keV, 40-keV images showed the highest overall objective and subjective quality scores. The CNRs for HCC and arteries in the arterial phase were 6.4 (IQR, 3.6–10.5) and 18.1 (IQR, 16.3–36.0), respectively; those for HCC and portal veins in the portal venous phase were 2.6 (IQR, 1.8–3.7) and 12.9 (IQR, 10.8–16.3), respectively. Subjective quality scores for image noise, vessel sharpness, and lesion conspicuity were 4 (IQR, 4–5), 5 (IQR, 5–5), and 5 (IQR, 4–5) in the arterial phase, and 4 (IQR, 4–5), 5 (IQR, 5–5), and 4 (IQR, 3–5) in the portal venous phase, respectively (Table S3).

Hence, 40-keV images were used for comparison with EID-CT images. Image quality and detection results are summarized in Table S4. VP-PCCT demonstrated significantly higher CNR for arterial-phase tumors compared with EID-CT (6.2 vs 2.3, *p* < 0.0001), with no significant differences observed in tumor SNR across contrast phases or in CNR for portal venous-phase tumors. The CNR of the portal vein was also higher with VP-PCCT relative to EID-CT (11. 9 vs 9.8, *p* = 0.01). The SNR of the arteries did not differ between groups. EID-CT showed higher CNR for the arteries (36.3 vs 21.2, *p* < 0.0001). In the portal venous phase, VP-PCCT yielded lower SNR (7.1 vs 12.1, *p* < 0.0001) and CNR (20.7 vs 29.2, *p* < 0.0001) for liver parenchyma compared with EID-CT.

VP-PCCT also provided better lesion conspicuity relative to EID-CT in the arterial (5 vs 4, *p* < 0.0001) and portal venous (4 vs 3, *p* < 0.0001) phases, despite higher image noise (4 vs 5 in both phases, both *p* < 0.0001). Using MRI for reference, VP-PCCT detected all lesions (103/103), whereas EID-CT missed two small lesions (5 and 6 mm in diameter). Tumor detection rates did not differ significantly between groups (*p* = 0.90).

### Lesion-based VP-PCCT perfusion analysis stratified separately by HCC histological grade and MVI status

Interobserver agreement was high for all perfusion parameters [ICC: 0.941 (95% CI: 0.913–0.960) to 0.997 (95% CI: 0.995–0.998)]. Hence, measurements from one reader were used for subsequent analyses. By histological grade, poorly differentiated lesions showed significantly higher tumor HPI (HPI_tumor_) compared with well-differentiated lesions (88.2% vs 77.7%, *p* = 0.02; Table 2), in addition to significantly higher BF and BV values in peritumoral liver and liver parenchyma regions, and significantly elevated FEP and PVP in liver parenchyma (all *p* < 0.05). All other perfusion parameter comparisons are shown in Table 2. By MVI status, MVI-positive tumors exhibited significantly lower tumor ALP (ALP_tumor_; 39.0 vs 44.4 mL/100 mL/min, *p* = 0.04), normalized BF of peritumoral region (BF_norm_peritumor_; 97.2% vs 108.4%, *p* = 0.04), and normalized FEP of peritumoral region(FEP_norm_peritumor_; 96.0% vs 103.8%, *p* = 0.04) compared with MVI-negative tumors. The remaining perfusion parameters showed no significant differences (Table 3).

**Table 2:** Lesion-based comparison of perfusion parameters in HCC, peritumoral region, and liver parenchyma stratified by HCC histological grade

Parameters	BF (mL/100 mL/min)	BV (mL/100 mL)	FEP (mL/100 mL/min)	ALP (mL/100 mL/min)	PVP (mL/100 mL/min)	HPI (%)
Grade group	Tumor					
Well-differentiated	69.3 (54.6–94.4)	7.62 (5.7–10.7)	50.1 (43.1–61.7)	41.3 (32.4–58.7)	15.1 (5.05–23.8)	77.7 (67.1–91.0)
Poorly differentiated	81.1 (63.2–97.7)	8.84 (6.0–11.1)	52.5 (40.9–61.7)	44.7 (30.5–58.4)	9.07 (4.12–19.6)	88.2 (80.3–93.1)
*p* value	0.18	0.43	0.75	0.75	0.12	**0.02**
Grade group	Peritumoral region					
Well-differentiated	32.7 (24.3–44.2)	1.94 (1.3–2.9)	31.1 (22.7–41.3)	9.63 (6.32–14.0)	68.2 (52.5–82.0)	12.9 (8.66–21.4)
Poorly differentiated	41.8 (29.6–50.3)	2.60 (2.0–3.3)	37.8 (23.3–44.7)	10.2 (8.17–13.6)	69.4 (46.9–82.4)	16.5 (9.15–37.9)
*p* value	**0.02**	**0.01**	0.19	0.14	1.00	0.12
Grade group	Liver parenchyma					
Well-differentiated	28.5 (19.5–41.1)	1.76 (1.20–3.10)	26.4 (18.6–39.4)	6.81 (4.82–10.4)	79.0 (62.2–89.6)	8.65 (5.97–13.7)
Poorly differentiated	41.2 (37.2–51.3)	3.14 (2.1–4.6)	39.6 (21.5–48.6)	7.99 (5.37–11.2)	89.0 (76.7–99.2)	7.85 (5.77–14.9)
*p* value	**0.003**	**0.002**	**0.02**	0.28	**0.01**	0.95
Grade group	Normalized peritumoral region^*^					
Well-differentiated	104.4 (85.3–159.9)	101.3 (71.6–165.8)	103.3 (88.2–151.8)	134.8 (104.8–196.5)	88.9 (75.6–104.6)	149.5 (106.3–220.0)
Poorly differentiated	103.5 (79.1–134.0)	99.3 (58.6–139.8)	100.2 (78.3–134.1)	141.6(109.4–224.8)	82.8 (55.3–93.9)	174.1 (116.5–311.2)
*p* value	0.43	0.46	0.28	0.85	0.12	0.28

Seventy-four well-differentiated HCCs (E-S grades I–II) were identified in 64 patients, whereas 29 poorly differentiated HCCs (grades III–IV) were identified in 28 patients

*HCC* hepatocellular carcinoma, *BF* blood flow, *BV* blood volume, *FEP* flow extraction product, *ALP* arterial liver perfusion, *PVP* portal venous perfusion, *HPI* hepatic perfusion index

^*^ Normalized peritumoral parameters were calculated as the ratio of peritumoral to corresponding liver parenchymal perfusion values and expressed as percentages

Statistically significant *p* < 0.05 values are in bold

**Table 3:** Lesion-based comparison of perfusion parameters in HCC, peritumoral region, and liver parenchyma stratified by MVI status

Parameters	BF (mL/100 mL/min)	BV (mL/100 mL)	FEP (mL/100 mL/min)	ALP (mL/100 mL/min)	PVP (mL/100 mL/min)	HPI (%)
Group	Tumor					
MVI (−)	74.7 (54.6–100.0)	7.77 (5.8–11.2)	51.5 (43.2–63.5)	44.4 (32.8–60.7)	12.9 (3.50–21.4)	82.5 (69.3–93.1)
MVI (+)	74.6 (56.9–84.3)	7.47 (5.8–9.8)	47.9 (36.7–56.3)	39.0 (28.3–44.7)	15.1 (8.89–23.1)	79.9 (69.9–88.5)
*p* value	0.59	0.28	0.15	**0.04**	0.30	0.39
Group	Peritumoral region					
MVI (−)	36.1 (25.2–48.1)	2.18 (1.5–3.2)	33.6 (23.7–42.9)	9.96 (7.7–14.1)	69.4 (52.7–86.9)	13.5 (9.0–21.6)
MVI (+)	28.8 (24.1–44.6)	2.11 (1.3–3.1)	24.2 (20.3–38.0)	9.60 (6.1–13.6)	59.0 (41.9–72.9)	14.8 (9.2–43.2)
*p* value	0.37	0.54	0.05	0.69	0.06	0.27
Group	Liver parenchyma					
MVI (−)	30.3 (20.0–45.5)	2.07 (1.2–4.0)	29.4 (18.7–41.4)	6.95 (4.82–10.4)	80.4 (62.2–92.3)	8.48 (5.7–13.0)
MVI (+)	37.8 (28.5–45.5)	2.34 (1.6–3.1)	33.9 (20.3–42.5)	7.70 (4.99–12.0)	76.4 (65.4–92.4)	10.8 (6.4–16.3)
*p* value	0.41	0.78	0.90	0.45	0.80	0.27
Group	Normalized peritumoral region					
MVI (−)	108.4 (87.4–160.9)	102.0 (71.3–166.4)	103.8 (88.6–160.1)	136.7 (103.4–198.2)	88.9 (75.3–103.6)	153.4 (104.5–232.5)
MVI (+)	97.2 (71.2–111.6)	89.5 (65.5–125.3)	96.0 (72.2–118.7)	125.0 (104.8–186.8)	76.5 (56.8–91.4)	158.5 (120.4–324.1)
*p* value	**0.04**	0.17	**0.04**	0.52	0.06	0.56

Eighty-one MVI-negative HCCs were identified in 72 patients, whereas 22 MVI-positive HCCs were identified in 20 patients. Normalized peritumoral parameters were calculated as the ratio of peritumoral to corresponding liver parenchymal perfusion values and expressed as percentages

*HCC* hepatocellular carcinoma, *MVI(+)* MVI-positive, *MVI(−)* MVI-negative, *BF* blood flow, *BV* blood volume, *FEP* flow extraction product, *ALP* arterial liver perfusion, *PVP* portal venous perfusion, *HPI* hepatic perfusion index

Statistically significant *p* < 0.05 values are in bold

### Logistic regression analysis of clinical characteristics and perfusion parameters associated with HCC histological grade and MVI status

Multivariable logistic regression confirmed peritumoral enhancement [OR, 6.86 (95% CI: 1.85–29.26); *p* = 0.005], HPI_tumor_ [OR: 1.06 (95% CI: 1.02–1.11), *p* = 0.009] and BF_peritumor_ [OR: 1.04 (95% CI: 1.00–1.09), *p* = 0.04] as independent risk factors for poorly-differentiated HCCs (Tables 4 and S5). Tumor diameter > 5 cm [OR: 4.89 (95% CI: 1.13–23.30), *p* = 0.04], ill-defined pseudocapsules [OR: 5.01 (95% CI: 1.47–19.80), *p* = 0.01], lower ALP_tumor_ [OR: 0.97 (95% CI: 0.93–1.00), *p* = 0.04]), and reduced BF_norm_peritumor_ [OR: 0.25 (95% CI: 0.05–0.82), *p* = 0.046] as independent risk factors for MVI-positive HCCs (Table 5 and S6).

**Table 4:** Univariate and multivariable logistic regression analysis of variables associated with HCC histological grade

Characteristic	Univariable logistic regression		Multivariable logistic regression	
	OR (95% CI)	*p* value	OR (95% CI)	*p* value
AFP (> 200 ng/mL)	3.20 (1.09–9.45)	0.03	2.80 (0.80–9.87)	0.10
Peritumoral enhancement	4.91 (1.64–15.5)	0.004	**6.86 (1.85–29.26)**	**0.005**
BF_tumor_ (mL/100 mL/min)	1.01 (1.00–1.02)	0.07	NA	NA
HPI_tumor_ (%)	1.04 (1.01–1.08)	0.02	**1.06 (1.02–1.11)**	**0.009**
BF_peritumor_ (mL/100 mL/min)	1.05 (1.02–1.08)	0.003	**1.04 (1.00–1.09)**	**0.04**
BV_peritumor_ (mL/100 mL)	1.22 (0.99–1.49)	0.06	NA	NA
ALP_peritumor_ (mL/100 mL/min)	1.07 (1.00–1.14)	0.05	NA	NA
HPI_peritumor_ (%)	1.03 (1.00–1.06)	0.04	NA	NA
BF_liver_ (mL/100 mL/min)	1.05 (1.02–1.08)	0.003	NA	NA
BV_liver_ (mL/100 mL)	1.38 (1.11–1.71)	0.004	NA	NA
FEP_liver_ (mL/100 mL/min)	1.04 (1.01–1.07)	0.02	NA	NA
PVP_liver_ (mL/100 mL/min)	1.03 (1.01–1.06)	0.01	NA	NA
PVP_norm_peritumor_ (%)	0.22 (0.04–1.16)	0.07	0.18 (0.02–1.13)	0.08

Only variables with *p* < 0.1 in univariable analysis were demonstrated in this table. NA (not applicable) indicates variables excluded from the final multivariable model by stepwise regression

Subscripts indicate measurement regions: _tumor = tumor, _peritumor = peritumoral liver parenchyma, _liver = remote non-tumoral parenchyma, _norm_peritumor = percentage of peritumoral to corresponding liver parenchymal perfusion values

*HCC* hepatocellular carcinoma, *AFP* α-fetoprotein, *BF* blood flow, *BV* blood volume, *FEP* flow extraction product, *ALP* arterial liver perfusion, *PVP* portal venous perfusion, *HPI* hepatic perfusion index

Statistically significant *p* < 0.05 values are in bold

**Table 5:** Univariate and multivariable logistic regression analysis of variables associated with MVI

Characteristic	Univariable logistic regression		Multivariable logistic regression	
	OR (95% CI)	*p* value	OR (95% CI)	*p* value
Barcelona Clinic Liver Cancer staging (B)	4.59 (1.25–17.0)	0.02	NA	NA
Maximum tumor diameter (> 5 cm)	3.27 (1.14–9.29)	0.03	**4.89 (1.13–23.30)**	**0.04**
Peritumoral enhancement	6.14 (1.98–19.7)	0.002	3.42 (0.82–14.70)	0.09
Pseudocapsule (ill-defined)	3.62 (1.37–10.3)	0.009	**5.01 (1.47–19.80)**	**0.01**
ALP_tumor_ (mL/100 mL/min)	0.97 (0.94–1.00)	0.03	**0.97 (0.93–1.00)**	**0.04**
FEP_peritumor_ (mL/100 mL/min)	0.96 (0.92–1.00)	0.06	NA	NA
PVP_peritumor_ (mL/100 mL/min)	0.98 (0.96–1.00)	0.07	NA	NA
HPI_peritumor_ (%)	1.03 (1.00–1.06)	0.06	1.04 (1.00–1.08)	0.09
HPI_liver_ (%)	1.04 (0.99–1.09)	0.09	NA	NA
BF_norm_peritumor_ (%)	0.35 (0.11–1.07)	0.07	**0.25 (0.05–0.82)**	**0.046**
FEP_norm_peritumor_ (%)	0.36 (0.12–1.13)	0.08	NA	NA

Only variables with *p* < 0.1 in univariable analysis were demonstrated in this table. NA (not applicable) indicates variables excluded from the final multivariable model by stepwise regression

Subscripts indicate measurement regions: _tumor = tumor, _peritumor = peritumoral liver parenchyma, _liver = remote non-tumoral parenchyma, _norm_peritumor = percentage of peritumoral to corresponding liver parenchymal perfusion values

*MVI* microvacular invasion, *BF* blood flow, *BV* blood volume, *FEP* flow extraction product, *ALP* arterial liver perfusion, *PVP* portal venous perfusion, *HPI* hepatic perfusion index

Statistically significant *p* < 0.05 values are in bold

### Between-group comparisons of independent perfusion risk factors

**Fig. 3:**
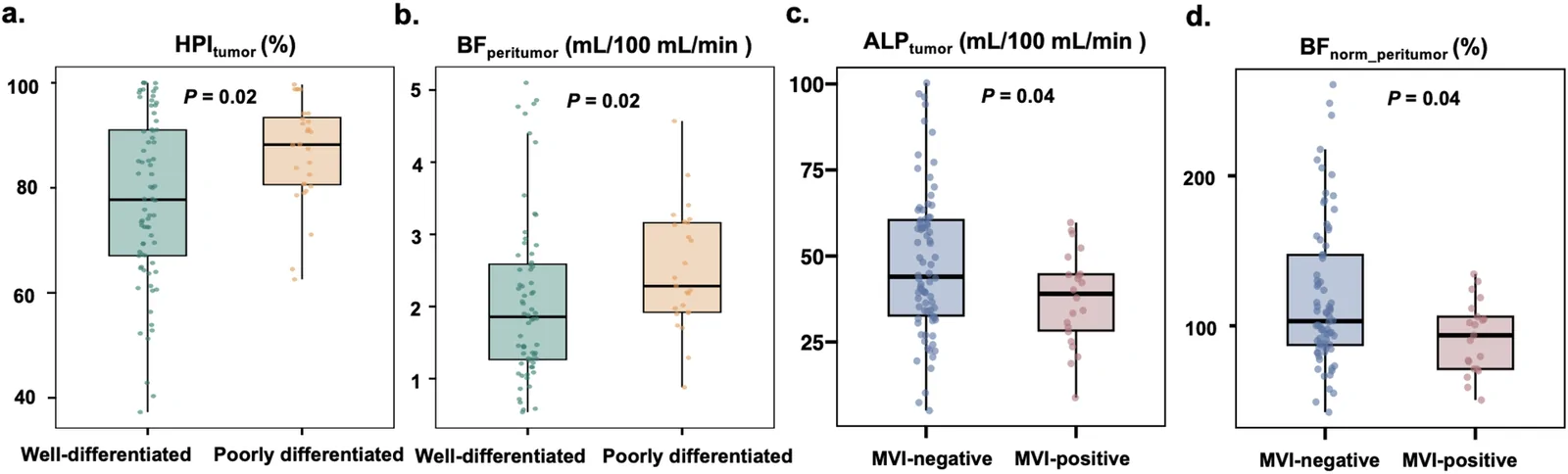
Boxplots showing the distribution of the independent perfusion-related risk factors associated with histological grade and MVI status in participants with HCCs. Poorly differentiated HCCs exhibited significantly increased HPI_tumor_ (**a**) and BF_peritumor_ (**b**) compared to well-differentiated tumors. Conversely, ALP_tumor_ (**c**) and BF_norm_peritumor_ (**d**) were significantly lower in MVI-positive compared to MVI-negative HCCs. In the box plots, the horizontal lines in the boxes indicate the median; the top and bottom lines of the boxes indicate the first and third quartiles, respectively. BF_peritumor_ = blood flow of peritumoral region, HPI_tumor_ = hepatic perfusion index of tumor, BF_norm_peritumor_ = percentage of peritumoral blood flow relative to corresponding liver parenchymal perfusion, ALP_tumor_ = arterial liver perfusion of tumor. MVI = microvascular invasion, HCC = hepatocellular carcinoma

**Fig. 4:**
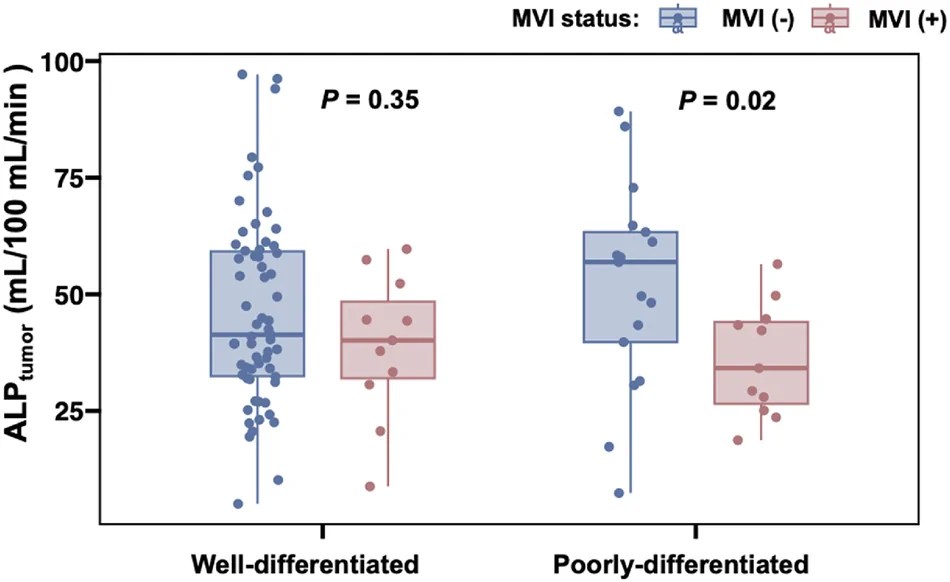
Subgroup analysis of ALP_tumor_ between HCCs with and without MVI. ALP_tumor_ was significantly lower in MVI-positive poorly-differentiated HCCs compared to their MVI-negative counterparts, whereas no significant difference was observed in well-differentiated HCCs. In the box plots, the horizontal lines in the boxes indicate the median; the top and bottom lines of the boxes indicate the first and third quartiles, respectively. ALP_tumor_, arterial liver perfusion of tumor; HCC, hepatocellular carcinoma; MVI, microvascular invasion

Comparisons of independent perfusion risk factors for histological grade and MVI status in HCCs are presented in Figs. 3 and 4. Poorly differentiated HCCs exhibited significantly higher HPI_tumor_ (88.2% vs 77.7%, *p* = 0.02) and BF_peritumor_ (41.8 vs 32.7 mL/100 mL/min, *p* = 0.02) compared with well-differentiated HCCs. MVI-positive HCCs showed lower ALP_tumor_ (39.0 vs 44.4 mL/100 mL/min, *p* = 0.04) and BF_norm_peritumor_ (97.2% vs 108.4%, *p* = 0.04) relative to MVI-negative HCCs. Subgroup analysis revealed that the ALP_tumor_ difference was significant in poorly-differentiated HCCs (57.4 vs 34.2 mL/100 mL/min, *p* = 0.02) but not in well-differentiated HCCs (41.7 vs 40.1 mL/100 mL/min, *p* = 0.35).

## Discussion

In HCCs, exhibiting heterogeneous recurrence and therapeutic response, tumor grade and MVI are key histologic predictors of aggressiveness and prognosis. However, the qualitative nature and interobserver variability of noninvasive morphological imaging limit its applicability. This study highlighted one-stop dynamic VVPCT with VP-PCCT as a viable option for HCC characterization. Extracted single-phase images offered image quality for lesion detection comparable to that of EID-CT at a lower radiation dose of 0.5 mSv per phase (13.3 mSv for 26 phases). Functionally, HPI_tumor_ and BF_peritumor_ were independent risk factors for poorly differentiated HCCs, whereas decreased ALP_tumor_ and BF_norm_peritumor_ were associated with MVI-positive HCCs. These findings support VP-PCCT as an alternative to multiphase EID-CT in HCC characterization, providing both sufficient morphological and functional data simultaneously in a single low-dose perfusion scan with a cumulative radiation dose of 13.3 mSv.

Although whole-organ VPCT shows promise for assessing tumor vascularization, detection, and characterization, its image quality is constrained by substantial noise from low-dose strategy acquisition [5]. Despite the low radiation dose per individual phase (0.7–1.25 mSv), the cumulative radiation dose remained relatively high (approximately 22.6–45 mSv) [7, 16–19]. Moreover, additional routine multi-phase CT is required to obtain sufficient morphological information for clinical diagnosis, further increasing the total cumulative exposure. Mean temporal images, created by averaging 3–5 dynamic datasets, could achieve noninferior image quality compared with 120-kVp images with cumulative doses of 14.5–27.6 mSv [20, 21]. However, such images are susceptible to motion-induced blurring. Conversely, in the current study, one-stop VP-PCCT generated individual phase images with comparable quality for HCC and vessel visualization while using a lower dose strategy (0.5 mSv per phase and 13.3 mSv for the full acquisition), compared with that in prior perfusion studies [7, 16–19]. Additionally, the single-phase dose in VP-PCCT was lower than that in the routine multi-phase EID-CT (0.5 mSv vs 4.7 mSv). These findings align with a prior pancreatic VP-PCCT study reporting high-quality morphologic imaging using individual phase images [14]. The performance improvement stems from PCCT’s inherent advantages, including radiation efficiency, elimination of electronic noise, and enhanced soft-tissue contrast resolution from low-keV reconstructions [8]. Moreover, single-phase reconstructions are less affected by motion artifacts, further improving lesion detectability. Thus, single-phase images from one-stop VP-PCCT provide sufficient image quality for HCC detection, avoiding the need for multiphase averaging or additional scans.

During HCC neoangiogenesis, intranodular portal supply decreases while arterial supply increases through the formation of unpaired arteries. Studies have demonstrated elevated ALP and HPI along with reduced PVP in HCCs compared to liver parenchyma [22–24]. Similarly, higher BF, BV, and permeability–surface area product were observed in HCCs compared to the background [25], likely reflecting increased tumor microvessel density [26, 27]. However, the relationship between histological grade and perfusion parameters remains debated. For instance, Sahani et al found that well-differentiated HCCs had higher BF, BV, and permeability compared with moderately or poorly differentiated tumors [25], whereas Ippolito et al observed no significant difference [22]. These inconsistencies may result from small cohorts and ROI selection, such as choosing the largest cross-section or solid component, given that high-grade tumors are often larger and necrotic. In this study of 103 lesions, HPI_tumor_ was higher in poorly differentiated HCCs than in well-differentiated tumors, consistent with the neoangiogenic process. Multivariable regression confirmed HPI_tumor_ and BF_peritumor_ as independent indicators of poorly differentiated HCCs, with BF reflecting perfusion efficiency and vascular supply. Nonetheless, further studies are needed to clarify the relationship between increased peritumoral vascular supply and histological grade.

MVI is a major prognostic factor for HCC recurrence postsurgery, defined as clusters of cancer cells within endothelial-lined vessels [28]. In the current study, correlations among MVI, radiological features, and perfusion parameters were examined. Consistent with previous studies [2], larger tumor size and ill-defined pseudocapsules correlated with MVI. Additionally, decreased ALP_tumor_ and BF_norm_peritumor_ were independent risk factors. Notably, MVI and histological grade displayed opposite perfusion patterns: poorly differentiated HCCs had increased HPI_tumor_, reflecting greater arterialization, whereas MVI-positive HCCs had lower ALP_tumor_ and BF_norm_peritumor_, indicating microvascular disruption. Wu et al reported higher PVP in MVI-positive HCCs relative to MVI-negative HCCs (108.1 vs 69.2 mL/min/100 mL, *p* = 0.0094), supporting the presence of hepatic artery-portal vein or portal vein-hepatic vein fistulas [29]. This mechanism was consistent with the decrease in ALP_tumor_ observed in the current study, although the association between ALP_tumor_ and MVI in poorly differentiated HCCs was based on a small subgroup and should be considered exploratory. Furthermore, the decrease in BF_norm_peritumor_ likely reflects impaired portal flow in the peritumoral regions, where MVI predominantly involves small portal vein branches, the liver’s major perfusion source (~ 75% of total BF) [30]. Together, decreased ALP_tumor_ and BF_norm_peritumor_ may potentially serve as hemodynamic markers of MVI risk.

This prospective study has some limitations. First, it was performed at a single center, and multi-center studies with larger sample sizes are needed to verify the robustness of the findings. Second, a direct intraindividual comparison of VP-PCCT and EID-CT was not conducted owing to radiation risk, with two scans per participant considered unfeasible; hence, PSM analysis was used to address potential confounding. Third, the optimal keV level was determined in a 20-patient subset of the VP-PCCT cohort, which may limit its generalizability. Patient-specific keV optimization was beyond the scope of this feasibility study and warrants further investigation. Fourth, as this study aimed to investigate the histological correlates of perfusion parameters in HCCs, all enrolled patients had surgically resectable tumors, mostly at an early stage. Future studies are warranted to assess the performance of this technique in patients with advanced stages to enhance generalizability. Fifth, CT perfusion parameters were not validated against histological markers, such as microvessel density. Therefore, further research is warranted to validate correlations between hemodynamic changes and histological characteristics. In addition, the subgroup finding that ALP_tumor_ differentiated MVI status only in poorly differentiated HCCs was based on a small sample size and requires independent validation. Finally, the diagnostic performance of perfusion parameters for noninvasive prediction of MVI and tumor grade was not evaluated and warrants further investigation.

## Conclusion

In conclusion, one-stop VP-PCCT offers sufficient image quality for clinical evaluation through single-phase images. HPI_tumor_ and BF_peritumor_ were independent indicators of poorly differentiated HCCs, whereas decreased ALP_tumor_ and BF_norm_peritumor_ predicted MVI-positive tumors. These findings indicate the feasibility of VP-PCCT for comprehensive HCC evaluation, including morphological assessment and hemodynamic profiling under low-dose conditions, but further studies are needed to confirm its clinical utility.

## Supplementary information

ELECTRONIC SUPPLEMENTARY MATERIAL

## Data Availability

The dataset used or analyzed during the current study is available from the corresponding author upon reasonable request.

## Abbreviations

ALP_tumor_: Arterial liver perfusion of tumor
BF_norm_peritumor_: Normalized blood flow of peritumoral region
BF_peritumor_: Blood flow of peritumoral region
BMI: Body mass index
CI: Confidence interval
EID-CT: Energy-integrating detector CT
HCC: Hepatocellular carcinoma
HPI_tumor_: Hepatic perfusion index of tumor
ICC: Intraclass correlation coefficient
MVI: Microvascular invasion
OR: Odds ratios
PCCT: Photon-counting detector CT
VMI: Virtual monoenergetic image
VP-PCCT: Dynamic volume perfusion CT with photon-counting CT
VPCT: Volume perfusion CT

## Declarations

### Methodology

- Prospective
- Observational
- Performed at one institution
