# Supplementary material for: Feasibility of one-stop multi-energy dynamic photon-counting perfusion CT for resectable HCC evaluation: lesion detection and histopathological correlations
Source: Insights Imaging. 2026 Nov 4;17:279. doi: 10.1186/s13244-026-02462-7 (PMC13737244; doi:10.1186/s13244-026-02462-7)
Supplement: Supplementary file 1 [file 13244_2026_2462_MOESM1_ESM.pdf]

Feasibility of One-stop Multi-energy Dynamic Photon-counting Perfusion CT for Resectable HCC Evaluation: Lesion Detection and Histopathological Correlations

ELECTRONIC SUPPLEMENTARY MATERIAL

Appendix S1: Inclusion and Exclusion Criteria

The inclusion criteria for the VP-PCCT cohort were: (a) adult participants and (b) suspected malignant focal liver lesions. The exclusion criteria for the VP-PCCT cohort were: (a) prior local ablation, transarterial chemoembolization, or systemic chemotherapy before the surgery, (b) pathologically confirmed non-HCC malignancies or presence of macrovasuclar invasion, (c) no surgical resection performed. The inclusion criteria for the retrospective EID-CT cohort were: (a) adult participants with pathologically confirmed HCC without macrovascular invasion, (b) not prior logical-regional HCC therapy or chemotherapy before surgery, (c) availability of both contrast-enhanced liver EID-CT examination and MRI within 1 month before surgery, and (d) availability of clinical laboratory data.

Appendix S2: Acquisition and scanning parameters for EID-CT

Three EID-CT systems from the same manufacturer (Philips Healthcare), including iCT, IQon Spectral CT, and Spectral CT 7500 scanners, were used for examinations. A non-ionic iodine contrast agent (Iomeron 400, 400mg I/mL; Bracco) was injected via an antecubital vein at a rate of 3 mL/s (1.2 mL/kg body weight), followed by 30 mL of normal saline solution. Late arterial-phase scans were automatically triggered using the bolus tracking technique with 17 seconds after the abdominal aorta reached 150 Hounsfield units, followed by portal-venous imaging 55 seconds later. Detailed scanning parameters for the three CT scanners are summarized in **Table S1**.

Appendix S3: Liver MRI Protocol

Liver MRI was performed with 1.5-T scanners (Magnetom Aera, Siemens Healthineers; uMR 660, United Imaging Healthcare) or a 3.0-T scanner (Ingenia, Philips Healthcare). The protocol included fat-suppressed T2-weighted imaging, diffusion-weighted imaging, unenhanced in- and out-of-phase T1-weighted imaging, and fat-suppressed 3D T1-weighted imaging before and after administration of extracellular contrast agents (arterial, portal venous, and delayed phases), in accordance with the technical recommendations of Liver Imaging Reporting and Data System Version 2018 [S1].

Appendix S4: VP-PCCT Image Postprocessing

A dedicated workstation(Syngo.Via; version VB60, Siemens Healthineers) was used for image postprocessing. The individual non-contrast, late arterial, and portal venous phases were extracted from dynamic PCCT perfusion imaging through Dynamic Angio application.

After automatic noise and body motion correction, three regions of interest (ROIs) were drawn on the aorta, portal vein, and liver to generate their time-attenuation curves (TACs). The first scan of the perfusion protocol served as the pre-contrast image. The late arterial phase was defined at the intersection of the aortic and portal venous TACs. The scan corresponding to the peak liver attenuation was considered the portal venous phase. Perfusion maps were generated using dedicated commercially available software (Body Perfusion, Syngo.Via, version VB60, Siemens Healthineers) after automatic four-dimensional noise and motion correction with non-rigid image registration, automatic bone removal, and vessel definition.

Appendix S5: Image Quality Assessment

The image quality of the late arterial phase and portal vein phase was compared between the VP-PCCT and EID-CT groups quantitatively and qualitatively. 1) **Objective image quality**: Circular ROIs were manually placed at the root of the celiac trunk, the main portal vein, liver parenchyma, the largest cross-sectional area of the lesion, and subcutaneous fat. The calcifications, plaques, and stenoses were avoided when placing vessel ROIs. Image noise was defined as the standard deviation (SD) of attenuation in subcutaneous fat. The signal-to-noise ratio (SNR) and contrast-to-noise ratio (CNR) were calculated as follows: $SNR_{tissue} = CT_{tissue}$ /image noise; $CNR_{vessel} = (CT_{vessel} - CT_{liver})$/image noise; $CNR_{lesion} = (CT_{lesion} - CT_{liver})$/image noise. 2) **Subjective image quality** [S2]: Qualitative assessments included image noise, visual lesion conspicuity, and vessel sharpness, each graded on a five-point scale. Image noise was graded as follows: 1 = unacceptable image noise, 2 = above-average increased noise, 3 = average image noise on an acceptable image, 4 = less-than-average noise, 5 = minimal or no image noise. Visual lesion conspicuity was assessed as: 1 = a not distinctly seen lesion, 2 = a suspicious lesion, 3 = a vaguely seen lesion with ill-defined margins, 4 = a well-seen lesion with poorly visualized margins, 5 = a well-seen lesion with well-visualized margins. Vessel sharpness was evaluated by the sharpness of the wall of the hepatic artery, portal vein, and their branches: 1 = blurry edges, 2 = worse than average, 3 = average, 4 = better than average, 5 = very sharp.

Appendix S6: ROI Delineation on Perfusion Maps

Perfusion parameters were assessed for tumor, peritumoral region, and liver parenchyma. For HCCs, ROIs were manually placed on the largest cross-sectional area of the lesion's solid component and on two adjacent slices, while avoiding necrotic and cystic areas. For the peritumoral region, crescent-shaped ROIs were drawn within a 1-cm rim surrounding the tumor, given that MVI predominantly occurs in peritumoral tissue and 1-cm threshold corresponds to established pathological grading criteria[S3]. ROIs were preferentially placed in the suspected enhancement area, defined as a grossly enhancing portion beyond the tumor contour on extracted late arterial phase images. In patients without a discernible hyperenhancing area, ROIs were placed randomly within the 1-cm peritumoral rim. For the liver parenchyma, standardized circular ROIs of 7 cm^2 were manually delineated in remote non-tumorous liver parenchyma at the level of the hepatic hilum, spatially separated from the tumor and excluding blood vessels and bile ducts. The mean across the three slices was used for analysis for all regions.

Appendix S7：Participant Characteristics

In the VP-PCCT cohort, a total of 315 patients were excluded for the following reasons: 179 had received prior tumor-related treatments (including local ablation, transarterial chemoembolization, or systemic chemotherapy) before surgery, 97 had pathologically confirmed non-HCC malignancies, 24 presented with macrovascular invasion, and 15 did not undergo surgical resection. Consequently, 92 patients were finally included in the study. The median interval between PCCT and MRI was 1 day (IQR 1–6), and between PCCT and surgery was 2 days (IQR 2–4).

Appendix S8：ROI Sizes

ROI sizes in HCCs ranged from 0.17 to 88.79 cm^2, depending on the extent of the solid tumor component. Peritumoral ROIs were delineated within a 1-cm rim surrounding the tumor, with sizes ranging from 0.22 to 12.13 cm^2 according to tumor size. Standardized circular ROIs of 7 cm^2 were delineated in remote non-tumorous liver parenchyma.

References

S1 Chernyak V, Fowler KJ, Kamaya A et al (2018) Liver Imaging Reporting and Data System (LI-RADS) Version 2018: Imaging of Hepatocellular Carcinoma in At-Risk Patients. Radiology 289:816-830

S2 Yu MH, Lee JM, Yoon JH et al (2013) Low tube voltage intermediate tube current liver MDCT: sinogram-affirmed iterative reconstruction algorithm for detection of hypervascular hepatocellular carcinoma. AJR Am J Roentgenol 201:23-32

S3 Edmondson HA, Steiner PE (1954) Primary carcinoma of the liver: a study of 100 cases among 48,900 necropsies. Cancer 7:462-503

Supplementary Tables

Supplementary Table 1: Liver Energy-integrating Detector CT Protocols and Reconstruction Parameters for Three Systems

Parameter / Modality	iCT	IQon Spectral CT	Spectral CT 7500
**Acquisition-related**			
**Manufacturer**	**Philips Healthcare**		
Tube voltage (kVp)	120	120	100
Spiral pitch	0.91	0.91	0.91
Rotation time (sec)	0.5	0.5	0.5
Collimation(mm)	128×0.625	64×0.625	128×0.625
Matrix size	512×512	512×512	512×512
**Reconstruction-related**			
Reconstruction algorithm	Iterative Model Reconstruction Level-1		
Filter	B		
Thickness (mm)	1	1	1
Increment (mm)	1	0.5	0.5

Supplementary Table 2: Clinical and Pathologic Characteristics of the Energy-integrating Detector CT Group

Characteristic	EID-CT group (n = 92)
Age (y)*	61 (51-69)
Body mass index (kg/m^2)*	23.3 (21.2-25.3)
Sex	
Male	76 (82.6)
Female	16 (17.4)
Etiology of liver disease	
Chronic hepatitis B/C	72 (78.3)
Others	20 (21.7)
Steatosis	2 (2.2)
Schistosomiasis	2(2.2)
Autoimmune hepatitis	1(1.1)
None	15 (16.3)
Child–Pugh classification	
A	90 (97.8)
B	2 (2.2)
Barcelona Clinic Liver Cancer stage	
Stage 0/A	88 (95.7)
Stage B	4 (4.3)
Laboratory findings	
α-fetoprotein level	
≤200 ng/mL	62 (67.4)
>200 ng/mL	30 (32.6)
Alanine aminotransferase level	
≤50 U/L	70 (76.1)
>50 U/L	22 (23.9)
Aspartate aminotransferase level	
≤40 U/L	51 (55.4)
>40 U/L	41 (44.6)
Tumor number per participant	
solitary	75 (81.5)
multiple	17 (18.5)
Tumor size	
≤5 cm	57 (62.0)
>5 cm	35 (38.0)
Pathologic findings	
Edmonson-Steiner grade (n_{lesion} = 104)‡	
I-II	64 (61.5)
III-IV	40 (38.5)
Microvascular invasion (n_{lesion} = 104)‡	
Positive	26 (25.0)
Negative	78 (75.0)
Inflammation grade of liver parenchyma	
G1-2	64 (69.6%)
G3-4	28 (30.4%)
Fibrosis stage of liver parenchyma	
S1-2	33 (35.9%)
S3-4	59 (64.1%)

Note.—Unless otherwise indicated, data are numbers of patients or tumors, and data in parentheses are percentages. *Data are medians, with interquartile ranges in parentheses.‡Number of lesions.

Supplementary Table 3: Image Quality Analysis Across Four Different Virtual Monoenergetic Image Energy Levels in the VP-PCCT derived individual phases images.

Phase	Parameter	kev Level	40-keV	50-keV	55-keV	60-keV	*p* value
**Quantitative Image Quality***							
Arterial Phase	SNR	HCC	6.6 (4.8–7.8)	7.2 (5.6–9.2)	7.3 (5.5–9.4)	6.0 (5.0–8.8)	0.05
	CNR	HCC	6.4 (3.6–10.5)[§, ‖, #]	5.1 (2.6–7.6)[‡, ‖, #]	4.4 (2.9–6.2)[‡, §]	2.7 (1.3–5.4)[‡, §]	**<0.001**
	SNR	Artery	23.4 (19.8–35.7)[#]	24.6 (17.4–31.6)[#]	20.6 (17.6–27.4)	22.3(14.9–25.0)[‡, §]	**0.04**
	CNR	Artery	18.1 (16.3-36.0)[‖, #]	16.7 (12.2–25.9)	13.0 (10.8–36.8)[‡]	16.2 (10.5–20.4)[‡]	**<0.001**
Portal Venous Phase	SNR	HCC	7.1 (4.9–8.5)[#]	7.4 (5.3–7.9)[#]	7.0 (5.9–7.9)[#]	5.5 (5.1–6.9)[‡, §,‖]	**0.03**
	CNR	HCC	2.6 (1.8–3.7)[§, ‖, #]	1.6 (1.2–3.4)[‡, #]	1.6 (0.8–2.6)[‡, #]	1.1 (0.4–1.8)[‡, §, ‖]	**<0.001**
	SNR	Liver	6.9 (5.9–8.2)	6.8 (6.2–8.1)	6.8 (6.4–7.7)	7.0 (6.8–7.8)	0.48
	CNR	Liver	21.5 (16.4–27.6)	18.9 (14.0–26.4)	18.3 (14.2–24.4)	16.6 (14.3–20.0)	0.06
	SNR	Portal vein	17.4 (13.1–	14.1 (12.2–19.3)[‡,]	12.2 (10.4–15.7)[‡,]	11.3 (9.0–13.5)[‡, §]	**<0.001**

			21.6)[§, ‖, #]	[‖, #]	[§]		
	CNR	Portal vein	12.9 (10.8–16.3)[§, ‖, #]	8.8 (7.5–10.9)[‡, #]	8.1 (6.0–9.8)[‡, #]	5.8 (4.1–7.6)[‡, §, ‖]	**<0.001**
**Qualitative Image Quality†**							
Arterial Phase	Image noise		4 (4–5)[#]	4 (4–5)[#]	4 (4–5)[#]	5 (5–5)[‡, §, ‖]	**<0.001**
	Vessels sharpness		5 (5–5)	5 (5–5)	5 (5–5)	5 (4–5)	0.40
	Lesion conspicuity		5 (4–5)[§, ‖, #]	5 (4–5)[‡, ‖, #]	4 (4–4)[‡, §]	4 (4–4)[‡, §]	**<0.001**
Portal Venous Phase	Image noise		4 (4–5)[#]	4 (4–5)[#]	4 (4–5)[#]	5 (5–5)[‡, §, ‖]	**<0.001**
	Vessels sharpness		5 (5–5)[#]	5 (5–5)[#]	5 (5–5)[#]	4 (4–5)[‡, §, ‖]	**<0.001**
	Lesion conspicuity		4 (3–5)[‖, #]	4 (3–4)[‖, #]	3 (3–4)[‡, §]	3 (3–4)[‡, §]	**<0.001**

Note.—VP-PCCT = dynamic volume perfusion CT with photon-counting CT, SNR = signal-to-noise ratio, CNR = contrast-to-noise ratio.
*Data are medians, with interquartile ranges in parentheses.
†Data are median scores, with range in parentheses.
‡Significant difference with 40-keV images.
§Significant difference with 50-keV images.
‖Significant difference with 55-keV images.
#Significant difference with 60-keV images.

Supplementary Table 4: Image Quality Comparison between VP-PCCT derived individual phase images and liver EID-CT images

Phase	Parameter		VP-PCCT	EID-CT	*p* value
**Quantitative Image Quality***					
Arterial Phase	SNR	HCC	5.5 (4.0–7.2)	5.2 (3.6–7.7)	0.82
	CNR	HCC	6.2 (5.5–7.6)	2.3 (1.9–2. 9)	**<0.0001**
	SNR	Artery	21.6 (15.3–26.3)	23.0 (15.0–32.7)	0.24
	CNR	Artery	21.2 (19.0–28.2)	36.3 (24.6–48.6)	**<0.0001**
Portal Venous Phase	SNR	HCC	5.7 (3.8–7.2)	6.3 (4.1–9.7)	0.07
	CNR	HCC	3.6 (1.8–6.9)	3.3 (1.3–5.4)	0.19
	SNR	Liver	7.1 (6.1–15.1)	12.1 (8.8–15.1)	**<0.0001**
	CNR	Liver	20.7 (16.9–24.0)	29.2 (23.4–40.6)	**<0.0001**
	SNR	Portal vein	15.2 (12.8–19.3)	15.4 (12.2–18.5)	0.67
	CNR	Portal vein	11.9 (10.5–12.4)	9.8 (6.7–10.8)	**0.01**
**Qualitative Image Quality**[†]					
Arterial Phase	Image noise		4 (3–4)	5 (5–5)	**<0.0001**
	Vessels sharpness		5 (4–5)	5 (4–5)	0.78

	Lesion conspicuity	5 (3–5)	4 (3–5)	**<0.0001**
Portal Venous Phase	Image noise	4 (3–4)	5 (5–5)	**<0.0001**
	Vessels sharpness	5 (4–5)	5 (4–5)	0.10
	Lesion conspicuity	4 (3–4)	3 (3–4)	**<0.0001**
**Detection rate**[‡]		103/103	120/122	0.90

Note.—VP-PCCT = dynamic volume perfusion CT with photon-counting CT, EID-CT = energy-integrating detector CT, SNR = signal-to-noise ratio, CNR = contrast-to-noise ratio.
[*]Data are medians, with IQRs in parentheses.
†Data are median scores, with range in parentheses.
[‡]The detection rate of the VP-PCCT group was derived based on dynamic enhancement CT images and HPI images.

Supplemental Table 5: Univariate Logistic Regression Analysis of Variables Associated with HCC Histological Grade

Characteristic	Univariable logistic regression	
	OR (95% confident intervals)	*p* Value
Age (>50 year)	0.51 (0.18–1.48)	0.21
Sex (female)	2.28 (0.72–6.99)	0.15
Chronic hepatitis history (present)	0.94 (0.37–2.48)	0.89
AFP (>200ng/mL)	3.20 (1.09–9.45)	**0.03**
ALT (>50 U/L)	0.89 (0.28-2.47)	0.82
AST (>40U/L)	1.35 (0.54-3.33)	0.51
BCLC staging (B)	1.99 (0.53-7.02)	0.30
Child Pugh grade (B)	2.58 (0.06-103)	0.56
Liver cirrhosis (present)	1.17 (0.47-3.00)	0.73
Maximum tumor diameter (>5 cm)	1.50 (0.53-4.04)	0.43
Tumor number (multiple)	0.83 (0.24-2.46)	0.75
Margin (nonsmooth)	1.85 (0.77-4.50)	0.17
Peritumoral enhancement	4.91 (1.64-15.5)	**0.004**
Pseudocapsule (ill-defined)	1.16 (0.47-2.80)	0.74
BF_{tumor} (mL/100 mL /min)	1.01 (1.00-1.02)	**0.07**
BV_{tumor} (mL/100 mL)	1.04 (0.95-1.15)	0.40
FEP_{tumor} (mL/100 mL/min)	1.00 (0.98-1.02)	0.78
ALP_{tumor} (mL/100 mL/min)	1.01 (0.99-1.03)	0.54
PVP_{tumor} (mL/100 mL/min)	0.98 (0.94-1.01)	0.24
HPI_{tumor} (%)	1.04 (1.01-1.08)	**0.02**
$BF_{peritumor}$ (mL/100 mL /min)	1.04 (1.01–1.07)	**0.02**
$BV_{peritumor}$ (mL/100 mL)	1.22 (0.99–1.49)	**0.06**
$FEP_{peritumor}$ (mL/100 mL/min)	1.03 (0.99–1.06)	0.14
$ALP_{peritumor}$ (mL/100 mL/min)	1.07 (1.00–1.14)	**0.05**
$PVP_{peritumor}$ (mL/100 mL/min)	1.00 (0.98–1.02)	0.99
$HPI_{peritumor}$ (%)	1.03 (1.00–1.06)	**0.04**
BF_{liver} (mL/100 mL /min)	1.05 (1.02–1.08)	**0.003**
BV_{liver} (mL/100 mL)	1.38 (1.11–1.71)	**0.004**
FEP_{liver} (mL/100 mL/min)	1.04 (1.01–1.07)	**0.02**

ALP_{liver} (mL/100 mL/min)	1.07 (0.97–1.17)	0.18
PVP_{liver} (mL/100 mL/min)	1.03 (1.01–1.06)	**0.01**
HPI_{liver} (%)	1.02 (0.98–1.06)	0.43
$BF_{norm\_peritumor}$ (%)	0.85 (0.43–1.68)	0.64
$BV_{norm\_peritumor}$ (%)	0.78 (0.45–1.34)	0.36
$FEP_{norm\_peritumor}$ (%)	0.86 (0.42–1.74)	0.67
$ALP_{norm\_peritumor}$ (%)	1.09 (0.76–1.56)	0.65
$PVP_{norm\_peritumor}$ (%)	0.22 (0.04–1.16)	**0.07**
$HPI_{norm\_peritumor}$ (%)	1.09 (0.88–1.35)	0.43

Note.—HCC = hepatocellular carcinoma, AFP = α-fetoprotein, ALT = alanine aminotransferase, AST = aspartate aminotransferase, BCLC = Barcelona Clinic Liver Cancer, BF = blood flow, BV = blood volume, FEP = flow extraction product, ALP = arterial liver perfusion, PVP = portal venous perfusion, HPI = hepatic perfusion index. Subscripts indicate measurement regions: _tumor = tumor, _peritumor = peritumoral region, _liver = liver parenchyma, _norm_peritumor = percentage of peritumoral to corresponding liver parenchymal perfusion values.

Supplemental Table 6: Univariate Logistic Regression Analysis of Variables Associated with HCC Microvascular Invasion

Characteristic	Univariable logistic regression	
	OR (95% confident intervals)	*p* Value
Age (>50 years)	0.41 (0.14-1.28)	0.12
Sex (female)	0.51 (0.07-2.07)	0.38
Chronic hepatitis history	1.57 (0.55-5.33)	0.42
AFP (>200 ng/mL)	1.55 (0.44-4.84)	0.48
ALT (>50 U/L)	0.50 (0.10-1.70)	0.29
AST (>40 U/L)	0.94 (0.32-2.55)	0.91
Barcelona Clinic Liver Cancer staging (B)	4.59 (1.25-17.0)	**0.02**
Liver cirrhosis	0.90 (0.32-2.54)	0.84
Maximum tumor diameter (>5 cm)	3.27 (1.14-9.29)	**0.03**
Tumor number (multiple)	1.31 (0.37-4.00)	0.65
Margin (nonsmooth)	2.02 (0.77-5.40)	0.15
Peritumoral enhancement	6.14 (1.98-19.7)	**0.002**
Pseudocapsule (ill-defined)	3.62 (1.37-10.3)	**0.009**
BF_{tumor} (mL/100 mL /min)	0.99 (0.98-1.01)	0.36
BV_{tumor} (mL/100 mL)	0.91 (0.80-1.04)	0.17
FEP_{tumor} (mL/100 mL/min)	0.98 (0.95-1.01)	0.11
ALP_{tumor} (mL/100 mL/min)	0.97 (0.94-1.00)	**0.03**
PVP_{tumor} (mL/100 mL/min)	1.02 (0.99-1.06)	0.17
HPI_{tumor} (%)	0.99 (0.96-1.02)	0.40
$BF_{peritumor}$ (mL/100 mL /min)	0.98 (0.95-1.02)	0.30
$BV_{peritumor}$ (mL/100 mL)	0.86 (0.65-1.15)	0.30

$FEP_{peritumor}$ (mL/100 mL/min)	0.96 (0.92-1.00)	**0.06**
$ALP_{peritumor}$ (mL/100 mL/min)	1.02 (0.95-1.09)	0.60
$PVP_{peritumor}$ (mL/100 mL/min)	0.98 (0.96-1.00)	**0.07**
$HPI_{peritumor}$ (%)	1.03 (1.00-1.06)	**0.06**
BF_{liver} (mL/100 mL /min)	1.01 (0.98-1.04)	0.45
BV_{liver} (mL/100 mL)	0.96 (0.75-1.22)	0.73
FEP_{liver} (mL/100 mL/min)	1.00 (0.97-1.03)	0.91
ALP_{liver} (mL/100 mL/min)	1.04 (0.93-1.15)	0.50
PVP_{liver} (mL/100 mL/min)	1.00 (0.98-1.02)	0.90
HPI_{liver} (%)	1.04 (0.99-1.09)	**0.09**
$BF_{norm\_peritumor}$ (%)	0.35 (0.11-1.07)	**0.07**
$BV_{norm\_peritumor}$ (%)	0.52 (0.23-1.14)	0.10
$FEP_{norm\_peritumor}$ (%)	0.36 (0.12-1.13)	**0.08**
$ALP_{norm\_peritumor}$ (%)	0.84 (0.54-1.33)	0.46
$PVP_{norm\_peritumor}$ (%)	0.35 (0.06-1.96)	0.23
$HPI_{norm\_peritumor}$ (%)	1.04 (0.82-1.32)	0.76

Note.—HCC = hepatocellular carcinoma, AFP = α-fetoprotein, ALT = alanine aminotransferase, AST = aspartate aminotransferase, BF = blood flow, BV = blood volume, FEP = flow extraction product, ALP = arterial liver perfusion, PVP = portal venous perfusion, HPI = hepatic perfusion index. Subscripts indicate measurement regions: _tumor = tumor, _peritumor = peritumoral region, _liver = liver parenchyma, _norm_peritumor = percentage of peritumoral to corresponding liver parenchymal perfusion values.
